# F2F-202, a Selective Histone Deacetylase 6 (HDAC6) Inhibitor, Behaves as an Arrow with Multiple Tips against Azole-Resistant *C. albicans*: Modulation of Yeast-to-Hyphae Transition, Trailing Effect, and Oxidative Stress

**DOI:** 10.1021/acsinfecdis.6c00195

**Published:** 2026-05-22

**Authors:** Simona Barone, Baptiste Mateu, Marialuisa Piccolo, Anna Guadagni, Carlo Irace, Francesca Lembo, Vincenzo Summa, Elisabetta Buommino, Margherita Brindisi

**Affiliations:** Department of Pharmacy, Department of Excellence 2023-2027, 478484 University of Naples Federico II, via D. Montesano 49, 80131 Naples, Italy

**Keywords:** *C. albicans*, chemoresistance, histone deacetylase 6, yeast-to-hyphae transition, Hda1, synergism

## Abstract

Fungal infections are increasingly threatening public health due to the onset of multidrug-resistant fungal strains. Accordingly, the incidence of chemoresistant *C. albicans* strains has steadily increased, along with associated mortality, thus highlighting the need for effective antifungal agents for the treatment of both localized and systemic *C. albicans* infections. Post-transcriptional modifications are key regulators of fungal virulence, modulating the production of virulence factors, yeast-to-hyphae transition, biofilm formation, and chemoresistance phenomena, whereas fungal histone deacetylases are key regulators of these processes. Herein, we report a potent and selective histone deacetylase 6 inhibitor (named F2F-202, **6**) showing synergistic activity in combination with the azole antifungal voriconazole (VRC) through the modulation of fungal histone deacetylase Hda1. Compound **6** showed growth inhibition against the azole-resistant *C. albicans* ATCC 10231 strain in combination with VRC, combined with a significant reduction of yeast-to-hyphae morphological transition. Moreover, the synergistic combination **6**/VRC affected the expression of key genes regulated by Hda1 activity and involved in morphogenesis, namely, *Nrg1*, *Als1*, and *Hpw1*. Additionally, **6** in combination with VRC was able to counteract the VRC-induced upregulation of *Erg11*, a gene involved in *C. albicans* azole resistance. Finally, our data suggest that the synergistic combination downregulates *Sod1* gene expression at an early point and impairs the antioxidant response. This study offers cues for the development of therapeutic options targeting resistant fungal infections and potentially overcoming the limitations associated with antifungal drugs currently in clinical use.

## Graphical abstract

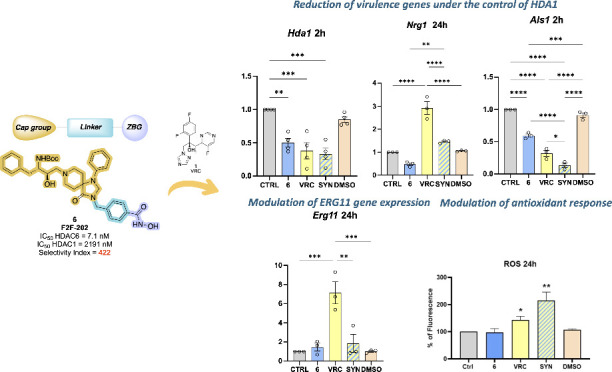

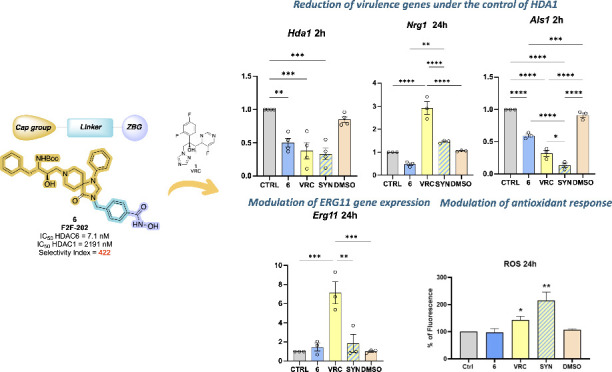

## Introduction

Fungal infections are arising as a public health burden due to the spread of invasive infections that are often difficult to diagnose and treat.^1,2^ Accordingly, the World Health Organization listed fungal priority pathogens for their clinical incidence on healthcare.^3^ Among critical priority fungal pathogens, *C. albicans* deserves special attention; it is a common opportunistic fungal pathogen that normally resides in the human gastrointestinal and genitourinary tracts and one of the most common causes of invasive fungal infections, thus posing a growing healthcare challenge due to its high morbidity and mortality, mainly in immunocompromised patients.^4,5^ Nowadays, few classes of drugs have been approved for the treatment of both systemic and superficial fungal infections. Among them are azoles (*e.g*., voriconazole (VRC), **1**, Figure 1), polyenes (*e.g*., amphotericin B, **2**, Figure 1), echinocandins (*e.g*., capsofungin, **3**, Figure 1), allylamines (*e.g*., terbinafine, **4**, Figure 1), and nucleoside analogues (*e.g*., flucytosine, **5**, Figure 1), which are classified according to their chemical structure and mechanism of action and represent the first-line treatment for both systemic and superficial mycoses.

**Figure 1:**
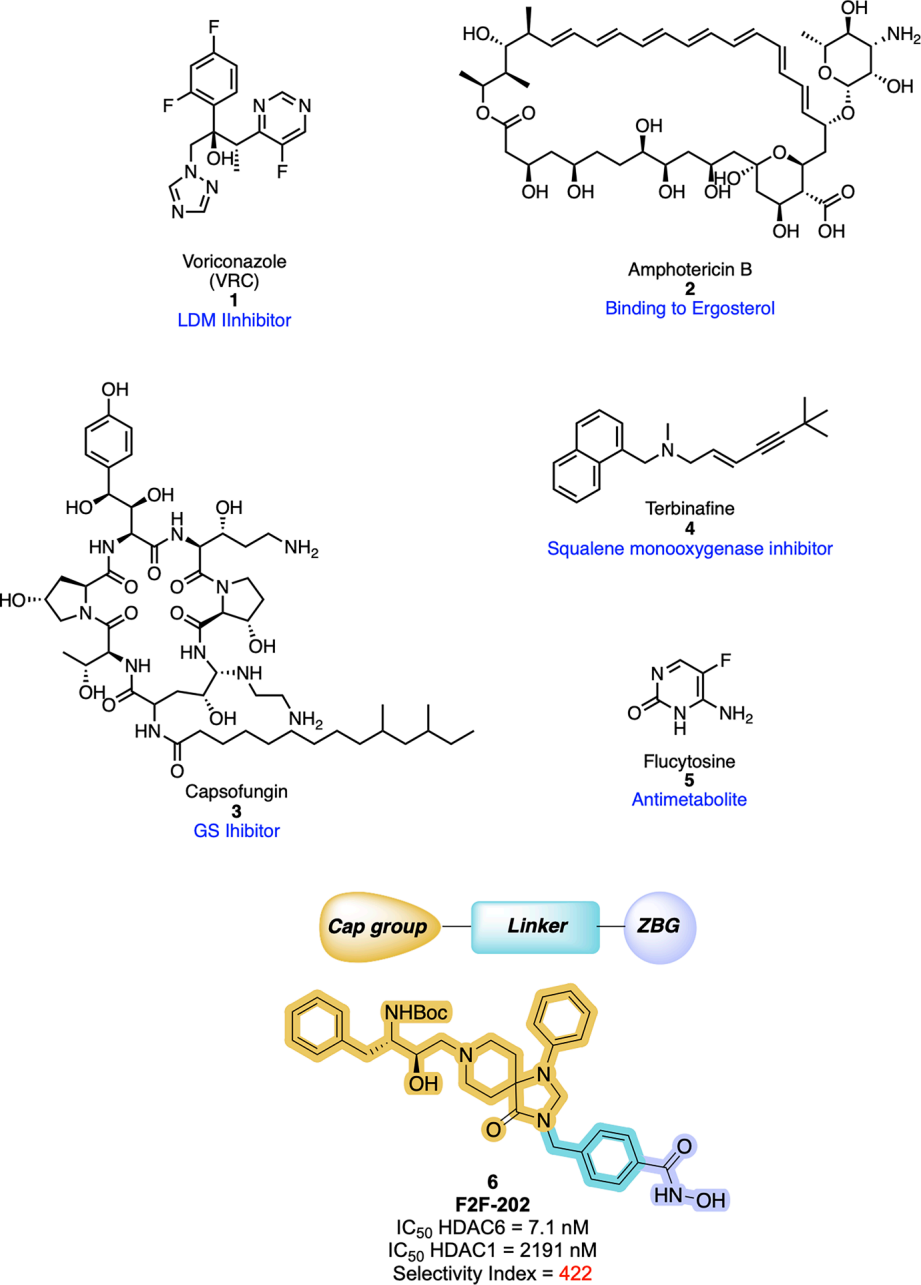
Representative structures of commonly used antifungal drugs **1**–**5** and their mechanism of action. LDM = 14α-sterol demethylase; GS = β-(1,3)-d-glucan synthase; General structure of HDACis, structure and potency of compound **6**. ZBG = zinc binding group.

Despite the broad-spectrum efficacy of these drugs in fighting fungal infections, they are associated with a wide range of adverse effects as well as poor pharmacokinetic properties. Most importantly, their abuse or misuse leads to chemoresistance phenomena, likely associated with the production of drug efflux pumps, target gene mutations, prodrugs inactivation, and yeast cell morphological changes, among others.^6–10^ In the past few years, novel noncanonical responses to azole drugs have been identified as the main cause of chemoresistance in addition to stable and genetically heritable mutations, namely, tolerance, persistence, and heteroresistance.^11^ For instance, a main limitation to azole treatment lies in their fungistatic activity, since surviving yeasts represent a reservoir for the development and selection of azole-resistant strains.^12^ Also, the trailing effect–usually observed during the treatment of *Candida* spp.–is a common tolerance phenomenon in which yeasts treated with azoles maintain a residual growth even at high antifungal concentrations, thus leading to full recovery of growth over time.^13^ The trailing effect is strictly influenced by environmental factors, including pH, temperature, and the concomitant use of different antifungal drugs, posing a relevant challenge in the treatment of phenotypic heterogeneous fungal infections.^14–20^ It has been demonstrated that the molecular chaperone heat shock protein 90 (Hsp90) is essential for the development and maintenance of antifungal resistance and tolerance, while it induces aneuploidy-mediated tolerance in *C. albicans* cells treated with miconazole.^21^ Moreover, external factors such as body temperature, pH, or CO_2_ levels can trigger the yeast-to-hyphae transition, whereas cells in hyphal form can express virulence factors that facilitate host tissue degradation and invasion and evade the immune system,^22–24^ where the thermal adaptation of fungi is driven by heat shock proteins.^25,26^ Notably, Hsp90 drives temperature-dependent morphological transitions in *C. albicans*, as reported by Robbins and Cowen.^27^ Mutations in the *Erg11* gene, which encodes for the azole target enzyme lanosterol-α-demethylase (LDM, involved in ergosterol biosynthesis) are also very common in *C. albicans*, representing the first cause of chemoresistance to azole drugs.^28–31^ Therefore, the treatment of fungal infections is posing significant healthcare challenges, which evidence the need for novel antifungals or antifungal adjuvants able to overcome chemoresistance phenomena and effectively manage both localized and systemic fungal infections. In this context, the growing evidence linking fungal virulence and growth to specific post-translational modifications (PTMs) (*i.e*., histone modifications) represents a crucial starting point for deciphering and counteracting resistance mechanisms.^32–36^

Based on structural homology with *S. cerevisiae* counterparts, 11 histone deacetylase (HDAC) isoforms have been identified in *C. albicans* and *C. glabrata* species, clustered into three main classes, listed below.^37–39^

1. Class I (Rpd3-type) includes RPD31, RPD32, HOS1, and HOS2 enzymes. RDP31 and RDP32, along with HOS1, have been found to be implicated in azole drug chemoresistance (*e.g*., trailing effect) and yeast filament extension,^40^ while HOS2 is mainly involved in regulating yeast-to-hyphae morphological transition,^41^ a peculiar phenomenon considered to be a key virulence trait of *C. albicans*, which can help fungi escape phagocytosis, increase dissemination during infection stages, and favor drug resistance phenomena.^42^ Although the role of HOS1 has not been fully elucidated, this enzyme is considered to mainly regulate cellular homeostasis and metabolism.^43^
2. Class II (HDA1-like) includes HDA1 and HOS3. *C. albicans* HDA1 is a close analogue of *S. cerevisiae* HDA1, which shares high sequence homology with Class II human HDACs.^44^ During yeast-to-hyphae transition, HDA1 is recruited to promoters of hypha-specific genes by the biofilm regulator 1 (BRG1) transcription factor, which in turn shapes chromatin in an unfavorable configuration for the binding with the transcriptional repressor NRG1, thereby resulting in hyphal development.^45^ HOS3 was first identified in 2001, although its biological role has remained poorly characterized.^50^
3. Class III includes sirtuins; among them, SIR2 was identified in *C. albicans* in 1999, and it exerts deacetylase activity on K16H4, thereby resulting in transcriptional repression.^46^
4. Other HDACs include SET3, which is a NAD^+^-dependent deacetylase. In its active form, SET3 assembles in a multiprotein complex, namely, the Set3 histone deacetylase complex (Set3C), which requires HOS1 deacetylase to exert its catalytic activity. Set3C is a key regulator of yeast-to-hyphae transition, modulates biofilm formation, and contributes to chemoresistance phenomena.^47^

Interestingly, the *HDA1* gene depletion in *C. albicans* cells inhibited hyphal development,^48^ thereby confirming that HDA1 is a key activator of this morphological transition. On the other hand, RPD31 can act as both an activator and repressor of yeast-to-hyphae transition. In yeast cells, RPD31 inhibits the expression of hyphal genes, while under filament-inducing conditions, it promotes filament elongation and hyphal gene expression.^49^ Accordingly, *C. albicans* strains defective for RPD31 showed reduced virulence *in vivo*.^50^ Likewise, genetic depletion of some Set3C components (*e.g*., SET3 and HOS2) induced hyperfilamentous phenotypes.^51^ In a similar manner to what happens in bacterial cells, yeasts can form biofilm to enhance their virulence, persistence in the host organism, and resistance to antifungal drugs.^52^ Both SET3 and HOS2 genetic depletions reduced biofilm formation,^47^ while key components of Set3C are repressed during cellular dispersion.^53^ Consistent with the enhanced virulence acquired by fungi during biofilm formation, HDA1 and RPD3 were found to be increased during azole-resistance acquisition, probably due to the increase in Hsp90 acetylation degree, and responsible for chemoresistance in fungi.^54,55^ In line with these data, both HDAC and Hsp90 inhibition were able to restore azole susceptibility in drug-resistant *C. albicans* strains.^56^ Furthermore, Hsp90 is a well-known deacetylation substrate for HDAC6 in humans, thus further highlighting their close cross-talk in biological processes.^57^

Given the strong epigenetic component underlying *C. albicans* virulence, HDACs have been investigated as potential therapeutic targets to counteract fungal pathogenicity. Accordingly, several human HDAC inhibitors (HDACis) displayed a weak antifungal activity alone, while significantly synergizing with azole drugs, thus representing a promising therapeutic strategy for the treatment of resistant *C. albicans* infections.^58–63^ More recently, several fungal HDACis showed synergistic efficacy with azole drugs in inhibiting morphological transition, biofilm formation, and yeast virulence,^64–66^ thereby paving a new avenue in the treatment of mycoses.

## Results and Discussion

### Compound **6** (F2F-202) Is a Novel, Highly Potent, and Selective HDAC6 Inhibitor

We recently disclosed a novel class of highly potent and selective HDAC6i by exploiting essential structural determinants to reach HDAC6 isoform selectivity, namely, (i) a bulky and rigid cap group, (ii) an aromatic linker, and (iii) a zinc binding group (ZBG) able to coordinate the catalytic zinc ion. Accordingly, we have also previously identified the *1-phenyl-1,3,8-triazaspiro­[4.5]­decan-4-one* motif as a suitable capping moiety for the preferential interaction with the HDAC6 catalytic surface over nuclear isoforms.^67–69^ Prompted by these results, we sought to evaluate the effect of more highly functionalized substituents on the piperidine nitrogen of the cap group. For this purpose, compound **6** (F2F-202), bearing a *tert*-butyl ((2*S*,3*R*)-3-hydroxy-1-phenylbutan-2-yl)­carbamate substituent on the nitrogen of the piperidine moiety, has been designed (Figure 1). This *smart handle* is a quite flexible structural moiety containing multiple points for polar (hydrogen bonding donors and acceptors) and hydrophobic interactions within the HDAC6 catalytic pocket, while potentially allowing the interaction with HDAC homologues from bacteria and fungi.^44,70^

The chemical synthesis of compound **6** is described in Scheme 1. The nucleophile amine **7** ^68^ has been exploited for the ring-opening reaction of commercially available (2*S*,3*S*)-*N*-*t*-Boc-3-amino-1,2-epoxy-4-phenylbutane, employing *i*PrOH as solvent, to provide the key synthetic intermediate **8**. The latter methyl ester was subsequently converted into the corresponding hydroxamic acid **6** upon treatment with 50% aqueous hydroxylamine in the presence of 4 M methanolic KOH as a base, with 25% yield.

**Scheme 1:**
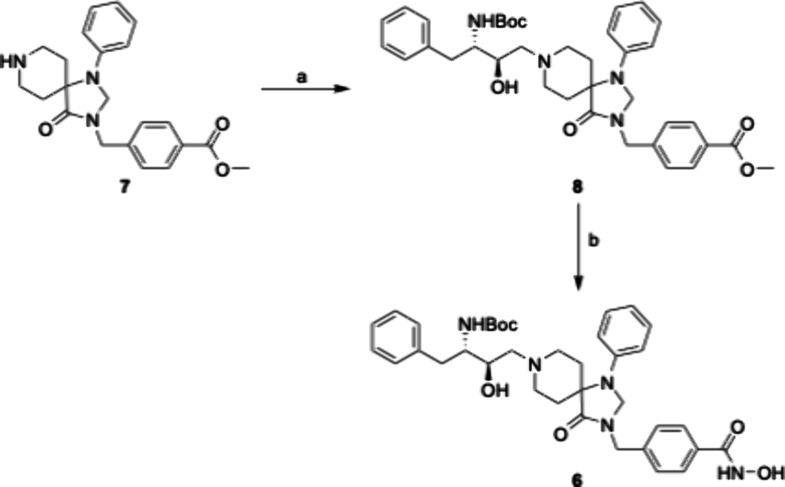
Synthesis of Compound **6** (F2F-202)^a^

a Reagents and reaction conditions: (a) (2*S*,3*S*)-*N*-*t*-Boc-3-amino-1,2-epoxy-4-phenylbutane or *tert*-butyl (*S*)-(oxiran-2-ylmethyl)­carbamate, *i*PrOH, 18 h, 80 °C, 66%; (b) 50% aq. NH_2_OH, 4 M KOH in MeOH, DCM/MeOH (2:1), 1 h, 25 °C.

Compound **6** has been assessed in enzymatic assays showing high potency on target protein HDAC6 (IC_50_ = 7.1 nM) with great selectivity over HDAC1 and selected as a representative of nuclear HDACs (IC_50HDAC1_ = 2192 nM; selectivity index 1/6 = 422). Building upon our previous results for the *in vivo* efficacy of selective HDAC6 inhibition in a murine model of *P. aeruginosa* infection, we recently assessed the efficacy of compound **6** in attenuating bacterial virulence and biofilm formation in *P. aeruginosa* PAO1 strains.^71^ Considering both the key role of fungal HDACs in controlling fungal virulence and the efficacy of proprietary HDAC6is in attenuating bacterial virulence and biofilm formation, we sought to also apply this concept to fungal infections and interrogate the ability of compound **6** in modulating fungal pathogenicity in *C. albicans* 90028 and *C. albicans* 10231 strains.

### Compound **6** (F2F-202) in Combination with VRC Inhibits *C. albicans* Cell Growth

Based on previous reports suggesting an important role of HDACis in controlling fungal virulence, we first assessed the effect of compound **6** on *C. albicans* 90028 and *C. albicans* 10231 cell growth. As expected, compound **6** alone did not significantly affect yeast cell growth in either strain, although it showed a moderate effect at 400, 200, and 100 μM on *C. albicans* 10231 with a percentage of growth reduction of 26%, 22%, and 12%, respectively (Figure 2). This result was in line with the reported weak intrinsic antifungal activity for HDACis.^72^

**Figure 2:**
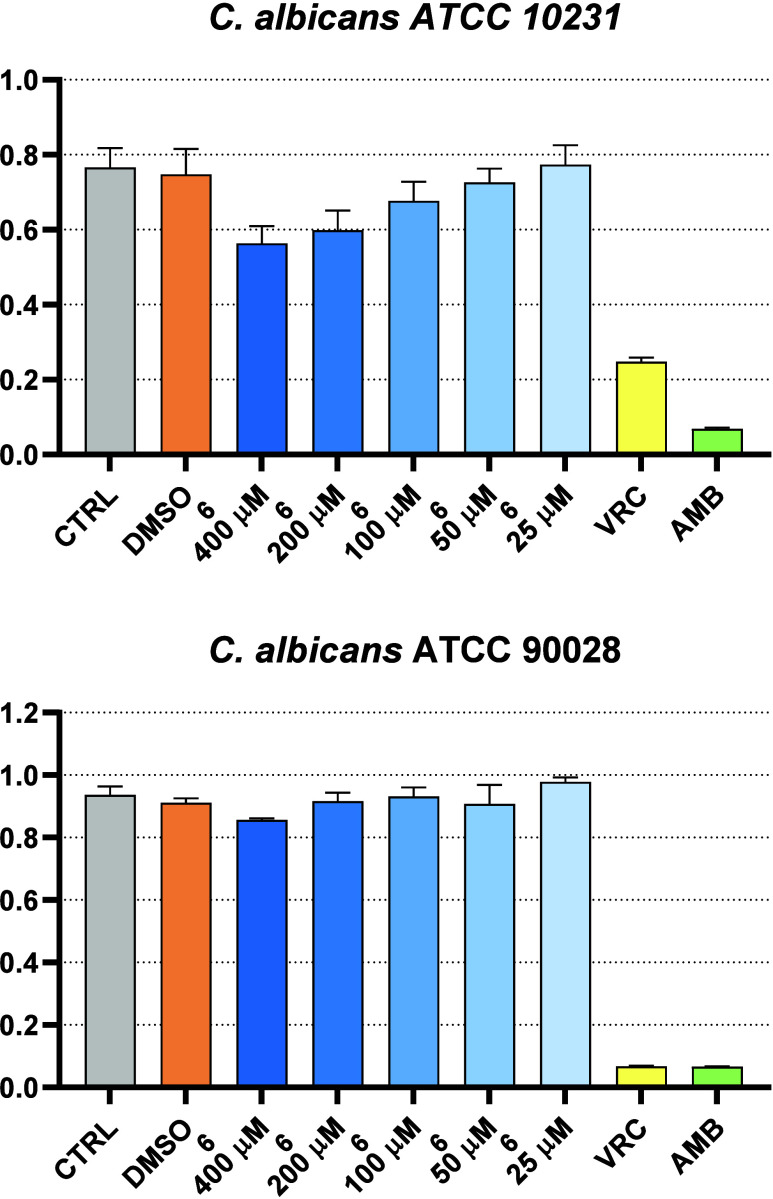
Cell growth inhibition assay on *C. albicans* 10231 (top) and 90028 (bottom) strains with compound **6** at different concentrations. VRC = Voriconazole 30 μg/mL for *C. albicans* ATCC 10231 and 0.125 μg/mL for *C. albicans* ATCC 90028. AMB = Amphotericin B.

In the next stage, since HDACis have been shown to enhance the efficacy of antifungals such as azoles, we investigated whether compound **6** could exert a synergistic effect in combination with VRC (**1**, Figure 1) on the *C. albicans* 10231 strain, which is resistant to VRC by itself. Compound **6** (100 μM) showed a fractional inhibitory concentration index (FICI) value of 0.129 in combination with VRC (0.125 μg/mL), indicative of a strong synergistic interaction. Additionally, the synergistic combination completely abrogated the yeast cell growth, compared to untreated and **6**-treated cells (Table 1). Conversely, VRC alone partially affected the cell growth of the resistant strain since, after 24 h of treatment, we observed a 58% reduction in *C. albicans* 10231 growth. However, VRC was no longer able to inhibit yeast growth after 48 h of treatment, while azole-resistant cell vitality was completely restored after 6 days of treatment. On the contrary, the synergistic interaction between compound **6** (100 μM) and VRC (0.125 μg/mL) completely inhibited cell growth after 6 days of treatment (Table 1). These data indicate that compound **6** in combination with VRC was able to inhibit *C. albicans* growth up to 24 h of treatment, and prolonged treatment (6 days) completely abrogated cell growth, reducing the possibility of acquiring resistance. Similarly, we conducted the same assay on the azole-susceptible *C. albicans* ATCC 90028 strain. Preliminary experiments were performed to establish the activity of VRC on *C. albicans* ATCC 90028 at different drug concentrations and time points. Consistent with literature evidence, lower VRC concentrations were associated with a more pronounced trailing effect (data not shown). Here, we showed that 0.125 μg/mL VRC was able to completely inhibit the growth of *C. albicans* ATCC 90028 after 24 h of treatment, while the drug lost its efficacy upon prolonged treatment (48 h or longer) (Table 1). These results were consistent with the occurrence of the trailing effect, a well-known phenomenon that is defined as a reduced but persistent growth of *C. albicans* at azole concentrations above the MIC.^13^ This is also in line with reported fungistatic activity of azoles upon prolonged treatment.^73^ On the contrary, the synergistic combination of compound **6** with VRC was fully efficacious against this strain, for all the days of treatment, contrasting the occurrence of the trailing effect.

**Table 1:** *C. albicans* ATCC 10231 and ATCC 90028 Growth at Different Time Points^a^

Time	Untreated *C. albicans* 10231	**6** 100 μM	VRC 0.125 μg/mL	**6**/VRC 100 μM/0.125 μg/mL	Untreated *C. albicans* 90028	**6** 100 μM	VRC 0.125 μg/mL	**6**/VRC 100 μM/0.125 μg/mL
0 h	3.47 ± 0.11	3.35 ± 0.21	3.32 ± 0.21	3.38 ± 0.28	3.47 ± 0.11	3.35 ± 0.21	3.32 ± 0.21	3.38 ± 0.28
24 h	7.00 ± 0.10	6.82 ± 0.12	4.06 ± 0.22	3.54 ± 0.28	7.20 ± 0.13	6.42 ± 0.11	3.86 ± 0.18	3.28 ± 0.25
48 h	7.18 ± 0.20	6.98 ± 0.15	7.95 ± 0.18	2.90 ± 0.19	7.39 ± 0.25	7.58 ± 0.09	6.98 ± 0.22	2.95 ± 0.29
72 h	8.30 ± 0.32	8.21 ± 0.24	8.02 ± 0.12	2.53 ± 0.12	8.01 ± 0.25	8.01 ± 0.14	8.24 ± 0.23	2.87 ± 0.22
6 d	8.42 ± 0.17	8.34 ± 0.11	8.18 ± 0.17	1.08 ± 0.25	8.12 ± 0.15	8.21 ± 0.01	8.58 ± 0.26	1.58 ± 0.22

a Yeast counts are given as means in log_10_ CFU/mL with standard deviations. Each experiment is the result of three independent experiments performed in triplicate. Untreated = Control cells that did not receive the treatment; VRC = voriconazole; **6**/VRC = synergistic combination.

### Compound **6** in Combination with VRC Inhibits *C. albicans* Yeast-to-Hyphae Transition

A key challenge in managing *C. albicans* infections is the ability of fungal cells to reversibly switch between unicellular yeast and hyphal growth forms in the host,^74^ where the filamentous form plays a pivotal role in the infection process since it can promote tissue penetration and escape from the immune system. To start the hyphal transcriptional program, the temporary removal of NRG1 protein is required. Multiple factors, such as the rise in temperature to 37 °C, are essential for the clearance of NRG1 and hyphal initiation. Accordingly, it was reported that inoculation of stationary cells into fresh medium at 37 °C was a powerful but transient inducer of hyphae.^75^ Accordingly, given the strong epigenetic component driving yeast phenotypic plasticity, in the next stage, we examined the morphological transition of *C. albicans* 10231 following treatments with compound **6**, VRC, or their synergistic combination after 2 and 24 h of treatment under hyphal induction conditions at 37 °C. As depicted in Figure 3A, control *C. albicans* exhibited the formation of elongated true hyphae after 2 h of observation with more pronounced elongation after 24 h, thereby confirming that yeast-to-hypha transition occurred (Figure 3F). On the contrary, hyphal elongation began by apical extension after 2 h of treatment with **6** (100 μM), VRC (0.125 mg/mL), or **6**/VRC combination, although the effect was less pronounced in comparison to the control group (Figure 3C–E). Interestingly, we did not observe a different morphology in the groups receiving compound **6** for 24 h where long hyphae were displayed (Figure 3H), while cells treated with VRC for 2 h showed shorter and sporadic hyphae compared to CTRL or DMSO (Figure 3I). Gratifyingly, the combination **6**/VRC led to a significant reduction in both the number and size of hyphae with a striking prevalence of yeast forms over hyphal ones (Figure 3J). The results were confirmed by acridine orange (AO) staining. Both control and treated cells showed a uniform green color after 24 h of treatment (Figure 3K–O). However, long hyphae were evident only in the groups receiving **6**, DMSO, or control, whereas short hyphae were present in the VRC group and absent in the group receiving **6**/VRC. Taken together, our results suggest that the synergistic interaction between compound **6** and VRC is able to inhibit fungal cell growth and effectively reduce the morphological transition to the hyphal phenotype.

**Figure 3:**
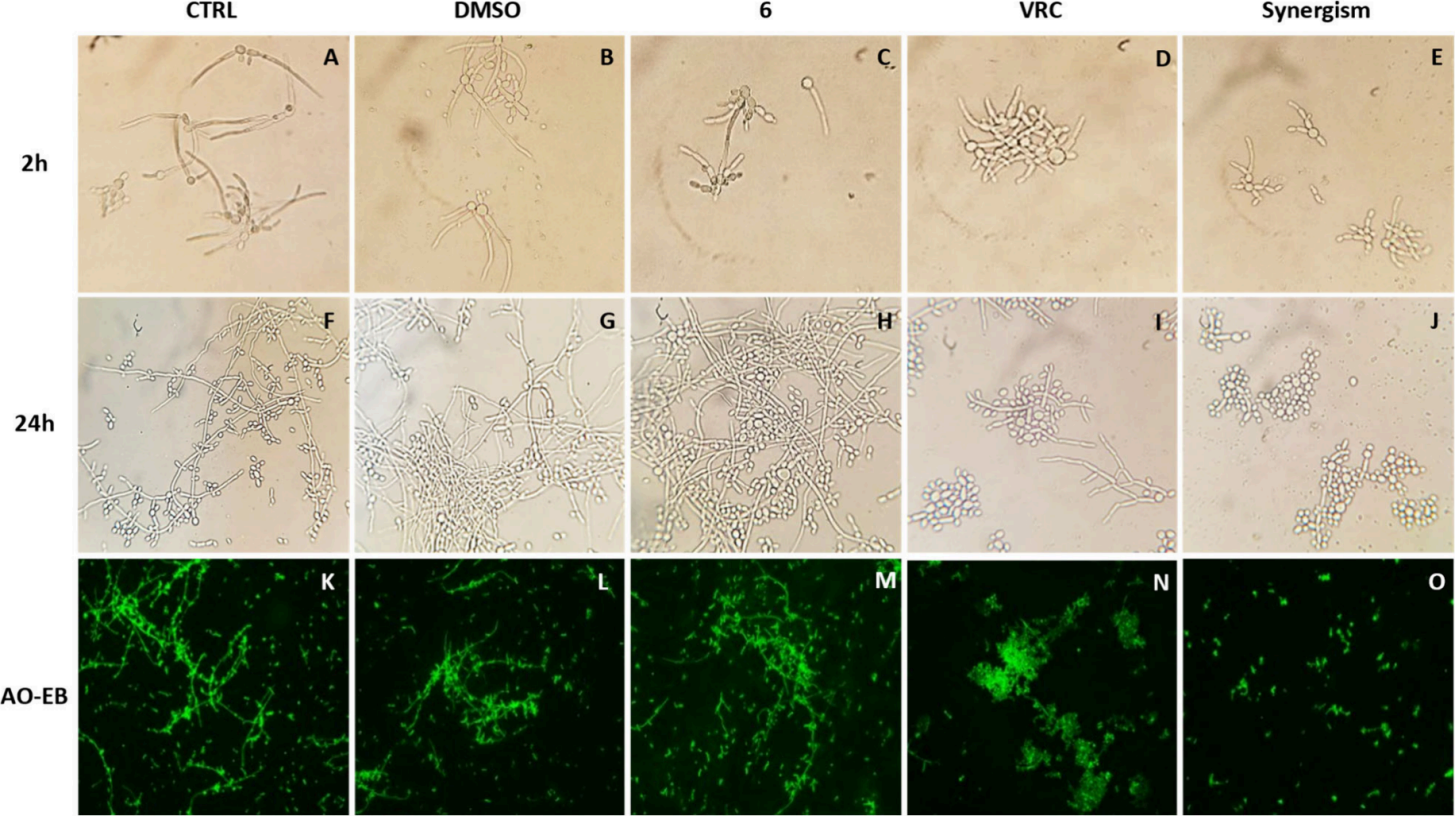
Effect of several treatments on the germination of *C. albicans* 10231 at 2 and 24 h. CTRL = no treatment; VRC, 0.125 μg/mL; **6**, 100 μM; Synergism: 100 μM **6** + 0.125 μg/mL VRC. Panels A–E: 2 h of treatment; panels F–J: 24 h of treatment. Observation under optical microscope with 400× magnification. Panels K–O: fluorescent microphotographs showing the yeast cell germination after 24 h of different treatments. Observed at 200× magnification (Iris Digita System, Twin Helix).

### Compound **6** in Combination with VRC Modulates the Expression of Genes Involved in Morphogenesis and Azole Resistance

To investigate the molecular mechanisms underlying the efficacy of the synergistic combination **6**/VRC, we first investigated the effect of compound **6** on the transcriptional expression of the *Hda1* gene in *C. albicans* 10231 upon 2 and 24 h of incubation with **6** alone or in combination with VRC (data not shown). As depicted in Figure 4A, *Hda1* gene expression was reduced after 2 h of treatment with compound **6** or VRC, either alone or in combination. Specifically, **6** induced a 50% decrease in *Hda1* gene expression, while VRC and the synergistic combination induced stronger reductions (62% and 64%, respectively). Conversely, 24 h of treatment with **6** alone or VRC induced a milder, nonsignificant reduction of *Hda1* expression compared with the control. The synergistic combination showed no significant change in the gene expression.

**Figure 4:**
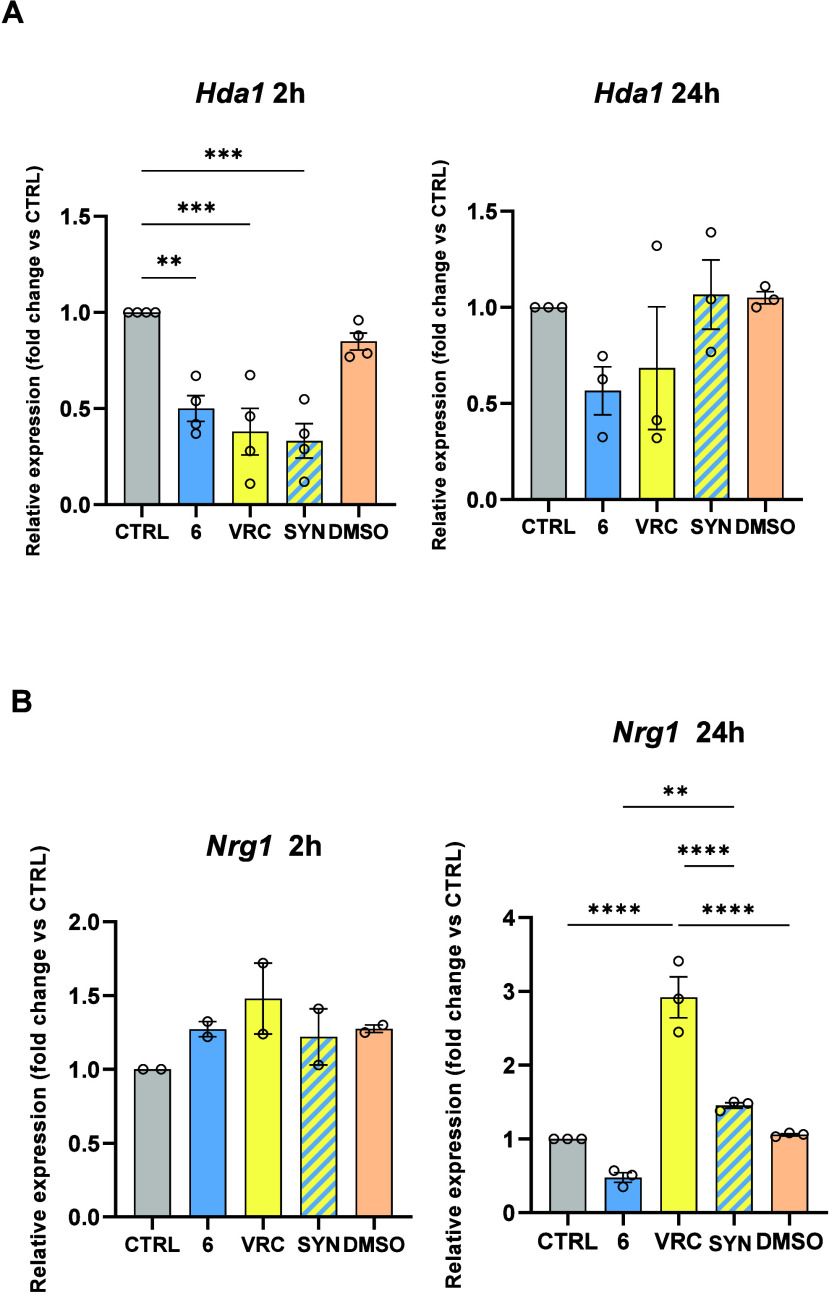
(A) Effect of *Hda1* gene expression at 2 and 24 h. CTRL = no treatment; VRC, 0.125 μg/mL; **6**, 100 μM; Synergism: 100 μM **6** + 0.125 μg/mL VRC. (B) Effect of **6** on *Nrg1* gene expression at 2 and 24 h. CTRL = no treatment; VRC (voriconazole), 0.125 μg/mL; **6**, 100 μM; Synergism: 100 μM **6** + 0.125 μg/mL VRC.

Taking into account the link between *Hda1* and the hyphal transcriptional repressor *Nrg1*,^76^ we analyzed whether **6** could be able to affect *Nrg1* expression, both alone and in combination with VRC. After 2 h of treatment, *Nrg1* gene expression tended to increase in all treated groups compared to controls, though not significantly (Figure 4B). The 24 h treatment with compound **6** alone induced significant changes in *Nrg1* gene expression relative to the control (52% reduction, *p* = 0.09), whereas VRC induced a marked increase in *Nrg1* gene expression (192%, Figure 4B). The combined treatment produced a moderate and not significant increase in *Nrg1* gene expression (45%, Figure 4B), suggesting an attenuated effect compared to VRC alone.

In the next stage, we analyzed the variation in gene expression of *Hwp1* and *Als1*, two key regulators of germination and virulence in *C. albicans*, under the negative transcriptional control of *Nrg1*.^45,76^ Agglutinin-like protein 1 (ALS1) is the major cell-surface adhesion protein that mediates both yeast-to-host tissue adherence and yeast aggregation.^77^ It binds glycans and mediates adherence to endothelial and epithelial cells, thus playing an important role in the pathogenesis of *C. albicans* infections.^78^ On the other hand, hyphal development promotes tissue penetration and escape from immune cells, while yeast cells can easily disseminate in the bloodstream. Consequently, the strong reduction of *Hwp1* gene expression indicates the suppression of factors promoting hyphal formation, impairing the invasive ability of yeast. Notably, as shown in Figure 5, both the *Hwp1* and *Als1* genes showed distinct expression profiles following treatment. *Als1* expression was significantly downregulated by both VRC and compound **6** after 2 h of treatment (**6**, 41% reduction; VRC, 68% reduction). The synergistic combination produced a much stronger effect, reducing *Als1* expression by 87% compared with the control. At 24 h, downregulation was less pronounced yet remained significant: VRC alone reduced expression by 56% and the combination, by 51%, whereas **6** alone had only a marginal, nonsignificant effect (−18%). Conversely, *Hwp1* displayed the opposite trend. At 2 h, expression was moderately reduced by compound **6** (29%) and more markedly by VRC (43%) with the combination yielding a comparable effect (44%). These reductions were not statistically significant. At 24 h, however, *Hwp1* expression was strongly suppressed by both VRC (−76%) and the synergistic treatment (−79%), while compound **6** alone had no significant impact.

**Figure 5:**
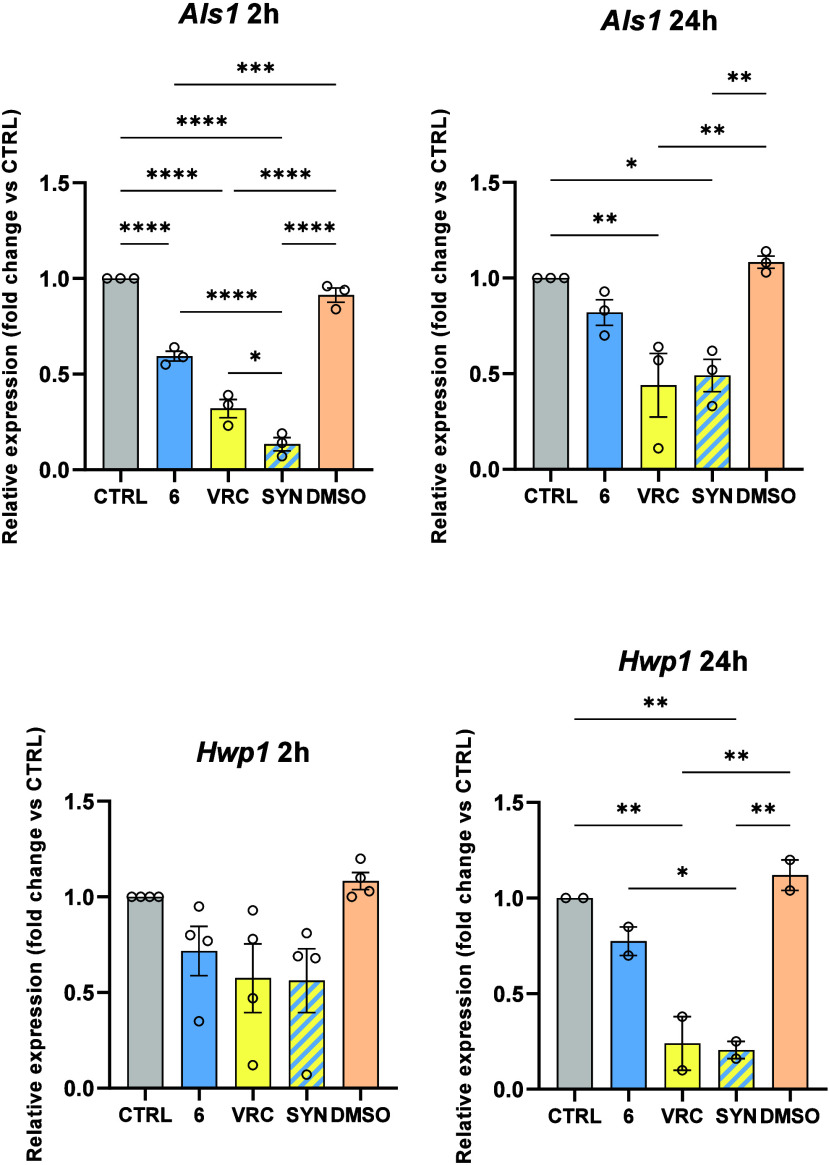
Effect of **6** on *Hwp1* and *Als1* gene expression at 2 and 24 h. CTRL = no treatment; VRC (voriconazole), 0.125 μg/mL; **6**, 100 μM; Synergism: 100 μM **6** + 0.125 μg/mL VRC. Left, top and bottom: 2 h treatment; right, top and bottom: 24 h treatment.

On the basis of these findings, it can be inferred that the synergistic combination **6**/VRC effectively inhibits yeast cells from the earliest stages of host interaction by downregulating the expression of *Als1*, an adhesin crucial for initial adhesion and biofilm development. At later stages, the synergistic treatment appears to act by suppressing the yeast-to-hypha transition, a key virulence factor of *C. albicans*, underlying its pathogenicity. The results are supported by morphological observations and real-time PCR data showing that, during the early hours, the synergistic combination is not particularly effective in inhibiting hyphal formation or the *Hwp1* gene expression; however, a pronounced reduction is observed after 24 h.

### Compound **6** in Combination with VRC Modulates the Expression of the Erg11 Gene

As previously mentioned, *Erg11* gene upregulation usually occurs in azole-resistant *C. albicans*;^79,80^ thereby, we have evaluated the effects of compound **6**, alone or in combination with VRC, on *Erg11* gene expression. Consistent with the literature evidence, we observed a 134% increase in *Erg11* gene expression after 2 h of treatment with VRC, whereas **6** alone did not show any significant effect. The synergistic combination partially attenuated the effect of VRC, resulting in a modest, nonsignificant 36% increase relative to the control (Figure 6). After 24 h, the VRC treatment induced a striking 613% increase in *Erg11* expression, consistent with a compensatory response to pharmacological stress and the activation of resistance mechanisms. The synergistic combination induced a modest, nonsignificant increase relative to the control but appeared to attenuate the effect of VRC more effectively.

**Figure 6:**
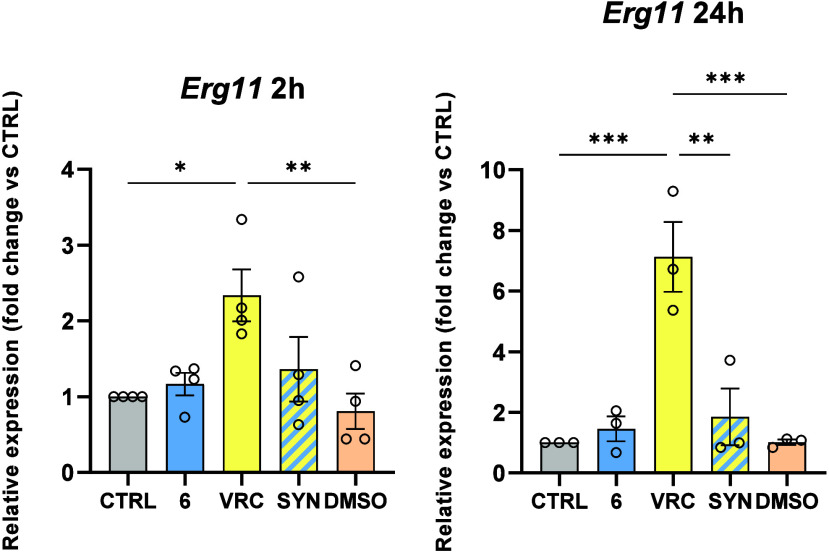
Effect of compound **6** on *Erg11* gene expression at 2 and 24 h. CTRL = no treatment; VRC (voriconazole), 0.125 μg/mL; **6**, 100 μM; Synergism: 100 μM **6** + 0.125 μg/mL VRC.

### Compound **6** in Combination with VRC Modulates the Antioxidant Response

Stress response pathways have been reported to be crucial for drug responses affecting tolerance.^81^ Consequently, combination therapy with new molecules that interfere with the compensatory stress response required for tolerance can potentiate the antimicrobial properties of the antifungal agents, thus reducing the risk of treatment failure.

To evaluate the response to VRC-induced stress in *C. albicans* 10231, we investigated the effect of compounds **6**, VRC, and their combination on the expression of the main genes involved in the oxidative stress response, *Sod1* and *Sod2*. After 2 h of treatment, compound **6** alone induced an increase in *Sod1* gene expression (Figure 7, 55% increase compared with the control), whereas VRC, which is known to act as a stress inducer for the yeast, was not effective in regulating *Sod1* expression. The synergistic combination **6**/VRC was, on the contrary, able to modulate *Sod1* expression (48% decrease) already after 2 h. At 24 h, though not significant, VRC induced a strong increase in *Sod1* gene expression (Figure 7, 146% increase), while **6** alone or its synergistic combination with VRC did not appear to influence gene expression. Exposure to DMSO, both at 2 and 24 h, did not result in relevant changes.

**Figure 7:**
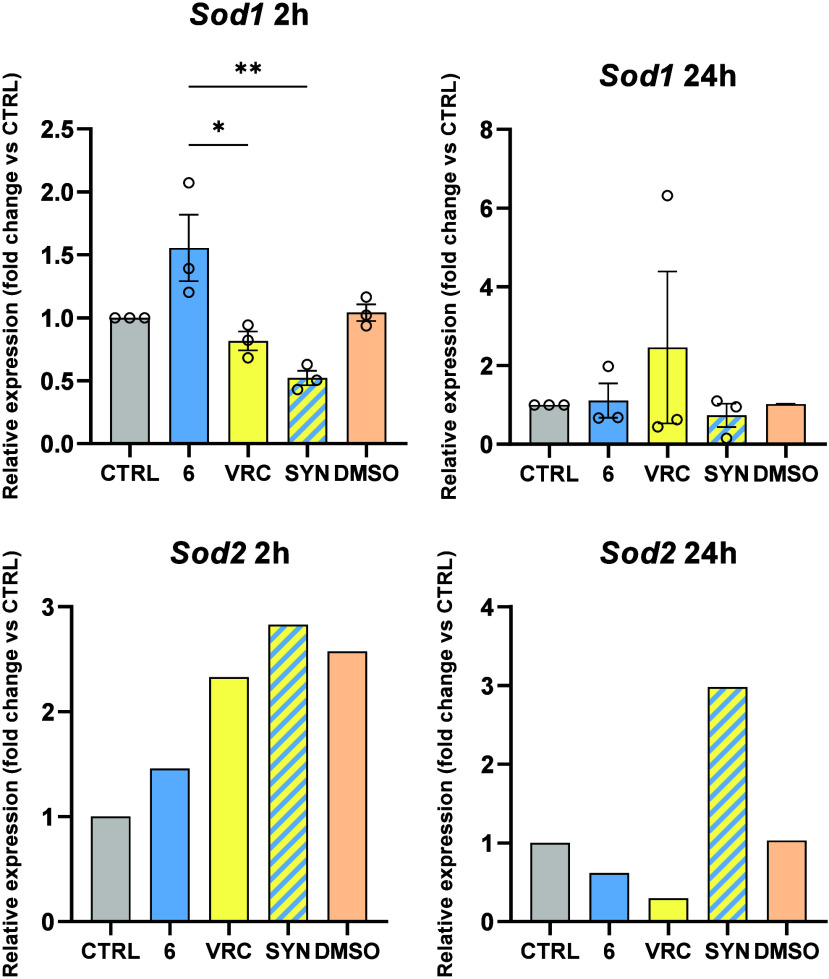
Effect of **6** on *Sod1* and *Sod2* gene expression at 2 and 24 h. CTRL = no treatment; VRC (voriconazole), 0.125 μg/mL; **6**, 100 μM; Synergism: 100 μM **6** + 0.125 μg/mL VRC.

These data are consistent with the results obtained from the ROS measurements. Specifically, at 24 h of treatment, molecule **6** alone did not cause any detectable increase in intracellular ROS levels. In contrast, VRC significantly induced ROS production, confirming its role as an inducer of the oxidative stress. Interestingly, the synergistic combination displayed a further significant increase in ROS level compared with the VRC-treated group, indicating an enhancement of oxidative stress in the presence of coexposure (Figure 8).

**Figure 8:**
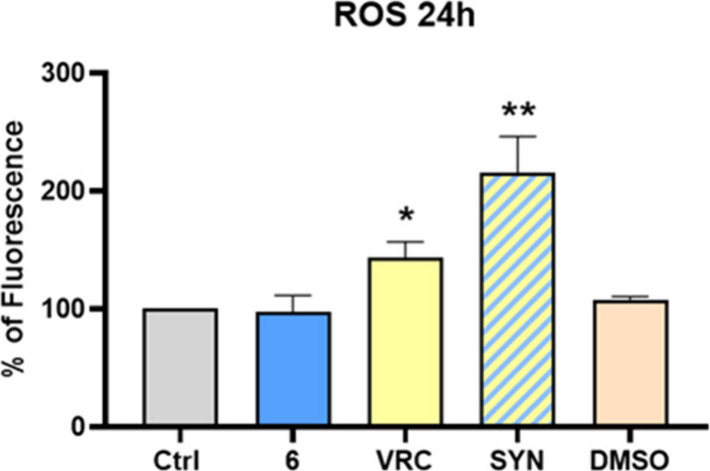
ROS production in *C. albicans*. Intracellular ROS levels evaluated by fluorescence analysis after 24 h of incubation with molecule **6**, Voriconazole (VRC), and their synergistic combination (SYN). DMSO-treated cells were used as negative control. Results are plotted in bar graphs as % of fluorescence compared to controls and are the mean of two independent experiments ± SEM (*n* = 6). *p < 0.05 with respect to control cells; **p < 0.01 with respect to control cells.

Overall, these data suggest that the synergistic combination **6**/VRC not only enhances ROS production but also downregulates *Sod1* at an early stage (2 h), indicating an impaired antioxidant response. This “double hit” effect may underlie the synergistic antifungal activity observed in *C. albicans*. Unlike *Sod1*, preliminary data for the *Sod2* gene indicate that it is not affected by all treatments, at either 2 or 24 h.

## Conclusions

Since the abuse or misuse of antifungals has led to the rapid emergence of multidrug-resistant yeast strains, nowadays, there is an urgent need for novel effective therapies to tackle both localized and systemic mycoses. Over the years, epigenetic mechanisms underlying invasive behavior in yeasts have been extensively studied, revealing that fungal HDACs are key regulators of yeast virulence, including morphological transitions, biofilm formation, and tissue dissemination. Accordingly, several HDACis have been assessed in *in vivo* infection models, showing a marked synergistic effect in combination with azoles, while a few examples of selective yeast HDACis have been reported so far. Considering the efficacy of proprietary HDAC6i compound **6** (internal code F2F-202) in counteracting *P. aeruginosa* virulence, we sought to also investigate its effect in *C. albicans* cells resistant to azoles. Overall, our results suggest that compound **6** may not exert its action exclusively through direct HDAC inhibition but rather may engage multiple mechanisms that deserve further investigation and clarification. Accordingly, compound **6** affected the expression of yeast HDAC homologue *HDA1*, leading to downregulation of *Als1* and *Hwp1* virulence-related genes, both under the negative transcriptional control of *Nrg1*. This effect was more pronounced when compound **6** was administered in combination with VRC, thereby suggesting a synergistic effect. Moreover, compound **6** was able to counteract *Erg11* gene upregulation induced by VRC when it was administered with VRC itself, suggesting that alternative pathways may be activated when compound **6** is used in combination with azole drugs. Also, compound **6** impaired the antioxidant response, thus suggesting that it may act on cellular stress response, potentially *via* the inhibition of Hsp90-related stress adaptation pathways, including azole exposure. Supportive of this hypothesis, the trailing effect observed in *C. albicans* strains sensitive to azoles testifies to the activation of Hsp90-dependent defense mechanisms to overcome the VRC therapeutic effect. Overall, our results validate compound **6** as an effective adjuvant compound that can synergize the effect of VRC. Our findings are also in line with previous studies reported for several HDACis, demonstrating their ability to inhibit morphological changes, cell growth, and azole-induced resistance, through the modulation of *Erg11* gene expression. Currently, we are further investigating molecular mechanisms underlying compound **6** efficacy in azole-resistant *C. albicans* strains.

## Experimental Section

### Chemistry

#### General Experimental Procedures

All reagents were used as received without further purification. NMR spectra were recorded on a Bruker Avance NEO 400, 700, or 600 MHz using residual solvent peaks as the reference. Experiments were carried out using CDCl3, DMSO-*d*_6_, or CD3OD as solvent at 25 °C with an RT-DR-BF/1H-5 mm-OZ SmartProbe. Compound purity was determined by integration of the diode array UV trace of UPLC chromatograms acquired on a Waters PDA UPLC system coupled with a single quadrupole. All analyses were conducted using a H_2_O/MeCN gradient containing 0.1% formic acid (FA). Elution was performed on an Acquity UPLC BEH C18 column (2.1 mm × 50 mm). The mobile phases were water with 0.01% formic acid (A) and acetonitrile with 0.01% formic acid (B). The elution gradient was performed according to the following conditions: 0–0.10 min, isocratic on 5% phase B; 0.10–2.60 min, linear gradient from 5% to 100% B; 2.60–2.90 min, isocratic on 100% B; 2.90–3.00 min, linear gradient to 5% B; 3.00–3.50 min, isocratic on 5% B for column recondition. The separation parameters were as follows: column temperature was set at 40 °C, inject volume was 10 μL, and the flow rate was set at 0.800 mL/min. Unless otherwise noted, all reactions were monitored by thin layer chromatography (TLC) on 5 × 20 cm sheets with 0.25 mm thickness (silica gel 60F254). Purification was carried out by liquid chromatography using 60 Merck silica gel (70–230 mesh ASTM). All compounds were determined to be >95% pure.

#### Methyl 4-((8-((2*R*,3*S*)-3-((*tert*-Butoxycarbonyl)­amino)-2-hydroxy-4-phenylbutyl)-4-oxo-1-phenyl-1,3,8-triazaspiro­[4.5]­decan-3-yl)­methyl)­benzoate (**8**)

To a stirring solution of *methyl 4-((4-oxo-1-phenyl-1,3,8-triazaspiro­[4.5]­decan-3-yl)­methyl)­benzoate* ^68^ (**7**, 0.207 mmol, 78 mg) in *i*PrOH (3 mL), *(2S,3S)-N-t-Boc-3-amino-1,2-epoxy-4-phenylbutane* (0.225 mmol, 59 mg) was added, and the resulting mixture was stirred at 80 °C. After 18 h, the solvent was evaporated under reduced pressure and the crude material was purified by silica gel liquid chromatography (DCM, DCM/MeOH 99:1 v/v), affording the desired product with 66% yield (88 mg).

MS (ESI) *m*/*z* calc. [M + H]^+^ for C_37_H_47_N_4_O_6_^+^, 643.35; found, 643.4.

^1^H NMR (700 MHz, CDCl_3_) δ 8.00–7.93 (m, 2H), 7.27 (m, 4H), 7.22–7.13 (m, 6H), 7.12 6.76 (m, 1H), 6.74 (d, *J* = 8.1 Hz, 2H), 4.91 (br, 1H), 4.57 (s, 2H), 4.49 (m, 3H), 3.90 (m, 1H), 3.78 (s, 3H), 3.71 (dd, *J* = 12.5, 3.3 Hz, 1H), 3.55 (m, 1H), 3.48 (dd, *J* = 12.5, 4.4 Hz, 1H), 3.15 (m, 1H), 2.93 (m, 1H), 2.87–2.76 (m, 3H), 2.75–2.43 (m, 4H), 1.64 (m, 2H), 1.30 (s, 9H).

#### *tert*-Butyl ((2*S*,3*R*)-3-Hydroxy-4-(3-(4-(hydroxycarbamoyl)­benzyl)-4-oxo-1-phenyl-1,3,8-triazaspiro­[4.5]­decan-8-yl)-1-phenylbutan-2-yl)­carbamate (**6**)

Compound **6** was obtained by starting from the corresponding methyl ester intermediate (0.137 mmol, 88 mg). To a stirring solution of **8**, a mixture of DCM/MeOH (2:1) and 50% aqueous hydroxylamine (100 equiv) was added, followed by methanolic 4 M potassium hydroxide (50 equiv). After 1 h, the reaction mixture was quenched by the addition of 6 N HCl and concentrated under vacuum. Crude material was purified by silica gel column chromatography (DCM/MeOH/NH_4_OH 20:1:0.05 v/v to 20:1:1 v/v), providing compound **6** with 25% yield (22 mg).

MS (ESI) *m*/*z* calcd [M + H]^+^ for C_36_H_46_N_5_O_6_^+^, 644.34; found, 644.4.

^1^H NMR (400 MHz, DMSO-*d*_6_) δ 11.19 (bs, 1H), 9.03 (bs, 1H), 7.75 (d, *J* = 6.4 Hz, 2H), 7.36 (d, *J* = 8.4 Hz, 2H), 7.27–7.15 (m, 6H), 6.87 (d, *J* = 8.1 Hz, 2H), 6.77 (t, *J* = 7.4 Hz, 1H), 6.63 (d, *J* = 9.0 Hz, 1H), 4.59 (d, *J* = 4.2 Hz, 4H), 3.71–3.58 (m, 2H), 3.00–2.92 (m, 1H), 2.85–2.73 (m, 4H), 2.68–2.53 (m, 3H), 2.46–2.31 (m, 2H), 1.60 (d, *J* = 12.4 Hz, 2H), 1.26 (s, 9H).

^13^C NMR (101 MHz, DMSO-*d*_6_) δ 173.62, 155.24, 142.84, 139.93, 139.48, 132.03, 129.15, 129.06, 127.87, 127.41, 127.35, 125.60, 118.00, 114.52, 77.29, 69.97, 62.65, 59.47, 55.13, 49.99, 49.67, 43.40, 35.32, 28.70, 28.22.

### Candida Strains and Culture Conditions

The yeast strains *C. albicans* ATCC 90028 (reference strain) and *C. albicans* ATCC 10231 (azole-resistant strain as reported in the ATCC “*C. albicans* drug resistance panel”, *Candida albicans* (Robin) Berkhout-10231|ATCC) were obtained from the American Type Culture Collection (Rockville, MD). Before each experiment, yeast cells were subcultured from the stocks onto Sabouraud dextrose agar (SDA) (Oxoid) at 30 °C for 48 h.

### Antifungal Susceptibility Testing

The antifungal activity of **6** was determined on *C. albicans* strains by using a standardized broth microdilution method.^82^ Briefly, the cell suspension was adjusted to 3 × 10^3^ CFU/mL in RPMI 1640 medium (Sigma) supplemented with 0.2% (w/v) glucose. One hundred microliter aliquots of these cell suspensions were dispensed into 96-well microtiter plates. Compound **6** was serially diluted using RPMI 1640 medium, and the mixtures were added to the wells at a final concentration ranging from 25 to 400 μM; the plate was incubated for 24 h at 37 °C. Amphotericin B (AMB, 2 μg/mL) and VRC (0.125 μg/mL for *C. albicans* ATCC 90028 and 30 μg/mL for *C. albicans* ATCC 10231) were chosen as the positive controls. The minimal inhibitory concentration (MIC) was defined as the lowest concentration of the compound that resulted in 100% growth inhibition after 24 h of incubation. Trailing was defined as the presence of residual yeast growth in all wells at supra-MIC concentrations.^83^ Voriconazole concentrations ranged from 0.06 to 32 μg/mL, and plates were incubated for 24 h at 37 °C. The trailing growth was assessed at 24 h, 48 h, 72 h, and 6 days. MICs and trailing measurements were performed in triplicates.^83^

### Checkerboard Assay

To investigate the interaction between **6** and VRC against *C. albicans* 10231, the checkerboard method in 96-well microtiter plates was used. Briefly, **6** and VRC were serially diluted along the *y* and *x* axes, respectively. The final concentrations ranged from 0.03 to 32 μg/mL (0.03, 0.06, 0.12, 0.25, 0.5, 1, 2, 4, 8, 16, 32) for VRC and from 25 to 200 μM (25, 50, 100, 200) for **6**. The checkerboard plates were inoculated with yeasts at an approximate concentration of 1 × 10^3^ CFU/mL and incubated at 37 °C for 24 h, following which yeast growth was assessed visually and the turbidity was measured by a microplate reader at 595 nm. The synergistic effect of the **6** and VRC combination was based on a calculation of the fractional inhibitory concentration index (FICI) for each drug pair. The fractional inhibitory concentration (FIC) for each combination was calculated as follows: FIC of $6=\frac{\mathrm{MIC of}\;6\;\mathrm{in combination}}{\mathrm{MIC of}\;6\;\mathrm{alone}}$ ; FIC of $\mathrm{VRC}=\frac{\mathrm{MIC of VRC in combination}}{\mathrm{MIC of VRC alone}}$ . The FICI is calculated by the sum of the FIC values for each drug. The FICI results for each combination were interpreted as follows: ≤0.5: synergistic; >0.5 to ≤1.0: additive; >1.0 to ≤2.0: indifferent; and >2.0: antagonistic effects.^84^ The minimum fungicidal concentration (MFC) was defined as the concentration that caused ≥3 log_10_ reduction in colony count from the starting inoculum plated on SDA incubated for 24 h at 37 °C. The MFC was determined by transferring 10 μL aliquots of the synergistic combination that yielded no fungal growth on agar plates and incubating the plates at 37 °C for 24 h. To evaluate the trailing effect, the CFU number was determined until 6 days of treatment. All the tests were conducted at least three times using independent cell suspensions.

### Effect of **6** on *C. albicans* Transition from Yeast to Hyphal Form

To determine the effect of **6** on the yeast-to-hypha transition, *C. albicans* ATCC 10231 (10^5^ cells) was grown in Sabouraud medium at 37 °C. **6** and VRC were added to the culture at 100 μM and 0.125 μg/mL, respectively, either alone or in combination for 2 and 24 h. Following incubation, the cultures were observed microscopically at 400× magnification and photographed to determine *C. albicans* morphology. Morphological transition was further analyzed by fluorescent microscopy with the acridine staining.^85^ Acridine orange (AO) is a commonly used dye that stains live cells in green fluorescence. Yeast cells were treated with **6** and VRC at synergistic concentrations as reported above. Cells were harvested by centrifugation after 24 h, stained with a solution of acridine orange and ethidium bromide (100 μg/mL), and kept in the dark for 30 min. Cells were harvested and washed twice with PBS to remove the excess stains. The cells were then observed at 20× magnification (Iris Digita System, Twin Helix).

### RNA Isolation and Real Time PCR

*C. albicans* was treated with 100 μM **6**, 0.06 μg/mL VRC, or a combination of both for 24 h. Total RNA was isolated by using the GenUp Total RNA kit (BiotechRabbit, Berlin, Germany) according to the manufacturer’s instructions. DNA contamination from the total RNA was removed by incubation with DNase I (RNase-free DNase Set, Qiagen, Hilden, Germany). Measuring the A260/A280 nm ratio assessed the nucleic acid purity. To generate cDNA, total RNA was reverse transcribed by using RevertUP II Reverse Transcriptase (BiotechRabbit, Berlin, Germany) into cDNA using random hexamer primers (Random hexamer, Roche Diagnostics, Monza, Italy) at 48 °C for 1 h according to the manufacturer’s instructions. Real time PCR was carried out using 1 μL of cDNA (5 ng/μL). Expression levels of all genes (see Table 2) were analyzed by quantitative real-time polymerase chain reaction (qRT-PCR) using the CFX96 system (Bio-Rad, Hercules, CA, USA) with the 2xSYBR Green qPCR Mix kit (GDSBio, Guangzhou, China). The thermal cycling parameters were as follows: activation of the DNA polymerase at 95 °C for 5 min; 40 cycles comprising 15 s at 95 °C for denaturation. For the annealing and extension temperatures, see Table 2. The housekeeping *Actin* (*Act*) gene was used as an internal control to normalize all data. The experiments were carried out in biological triplicate and in technical duplicate for each data point. Statistical analysis was performed using one-way ANOVA followed by Tukey’s post hoc test. For all graphs, data are presented as mean ± SEM with individual data points overlaid.

**Table 2:** Primer Sequences Used for the qRT-PCR and Annealing Conditions

Gene	Sequence (5′–3′)		Annealing and extension
*ERG11*	Fw	ACCATTTGGTGGTGGTAGACA	55 °C
	Rev	AGGGTCAGGCACTTTATAACCA	
*TUP1*	Fw	CTCTTGGCGACAGGTGCAG	59 °C
	Rev	GTGGTGACGCCGTCTTCGA	
*NRG1*	Fw	CACCTCACTTGCAACCCC	59 °C
	Rev	GCCCTGGAGATGGTCTGA	
*HWP1*	Fw	TGGTGCTATTACTATTCCGG	59 °C
	Rev	CAATAATAGCAGCACCGAAG	
*HDA1*	Fw	GCACGACGGTGATTATGTTTATG	56 °C
	Rev	GCAGCATCAAATCCAGAACTAAC	
*ALS1*	Fw	TGCCATATCATACTACCACAACTG	56 °C
	Rev	CAGTTGGATTTGGCAGTGG	
*ACT*	Fw	CCAGCTTTCTACGTTTCC	54 °C
	Rev	CTGTAACCACGTTCAGAC	
*SOD1*	Fw	TTGAACAAGAATCCGAATCC	55 °C
	Rev	AGCCAATGACACCACAAGCAG	
*SOD2*	Fw	ATGTTTTCTATCAGATCATC	55 °C
	Rev	ACCACCACCTTGAGAGACAGGAGCC	

### *In Vitro* ROS Detection

Cellular ROS production was assessed by using the ROS Detection Assay Kit (Canvax Biotech, Valladolid, Spain) based on dichlorodihydrofluorescein diacetate (H2DCFDA)—a fluorogenic dye that detects intracellular hydroxyl radicals, peroxides, and other reactive oxygen species. Operatively, *C. albicans* was seeded in a black 96-well plate and cultured at 37 °C with 5% CO_2_ for 24 h. Then, cells were treated or not with VRC (0.125 μg/mL), molecule **6** (100 μM), and their synergistic combination (SYN) for 24 h. At the end of treatments, cells were resuspended in fresh medium containing H_2_DCFDA working solution (25 μM). After 1 h of incubation, the fluorescence intensity was measured at Ex/Em = 485/530 nm using a fluorescence microplate reader (Promega, GloMax, Madison, WI, USA). The vehicle DMSO was used as negative control.

## Supplementary Material



## Notes

^1^H and ^13^C NMR spectra of **6** (F2F202); UPLC-mass spectra of **6** (F2F202); *In vitro* bioscreens of **6** (F2F202) (PDF)
