# Supplementary material for: F2F-202, a Selective Histone Deacetylase 6 (HDAC6) Inhibitor, Behaves as an Arrow with Multiple Tips against Azole-Resistant C. albicans: Modulation of Yeast-to-Hyphae Transition, Trailing Effect, and Oxidative Stress
Source: ACS Infect Dis. 2026 May 22;12(6):2101–13. doi: 10.1021/acsinfecdis.6c00195 (PMC13270531; doi:10.1021/acsinfecdis.6c00195)
Supplement: Supplementary file 1 [file id6c00195_si_001.pdf]

SUPPORTING INFORMATION

F2F-202, a selective histone deacetylase 6 (HDAC6) inhibitor, behaves as an arrow with multiple tips against azole-resistant *C. albicans*: modulation of yeast-to-hyphae transition, trailing effect, and oxidative stress.

Simona Barone,[a] Baptiste Mateu,[a] Marialuisa Piccolo,[a] Anna Guadagni,[a] Carlo Irace,[a] Francesca Lembo,[a,] Vincenzo Summa,[a] Elisabetta Buommino,[a,*,#] and Margherita Brindisi[a,*,#]*

[a] Department of Pharmacy, Department of Excellence 2023-2027, University of Naples Federico II, via D. Montesano 49, 80131, Naples, Italy

# co-last authors

* Correspondence: margherita.brindisi@unina.it elisabetta.buommino@unina.it francesca.lembo@unina.it

Table of Contents

^{1}H and ^{13}C NMR spectra of **6 (F2F202)** S2

UPLC-mass spectra of **6 (F2F202)** S3

Bioscreens *in vitro* S4-S5

^{1}H NMR of 6

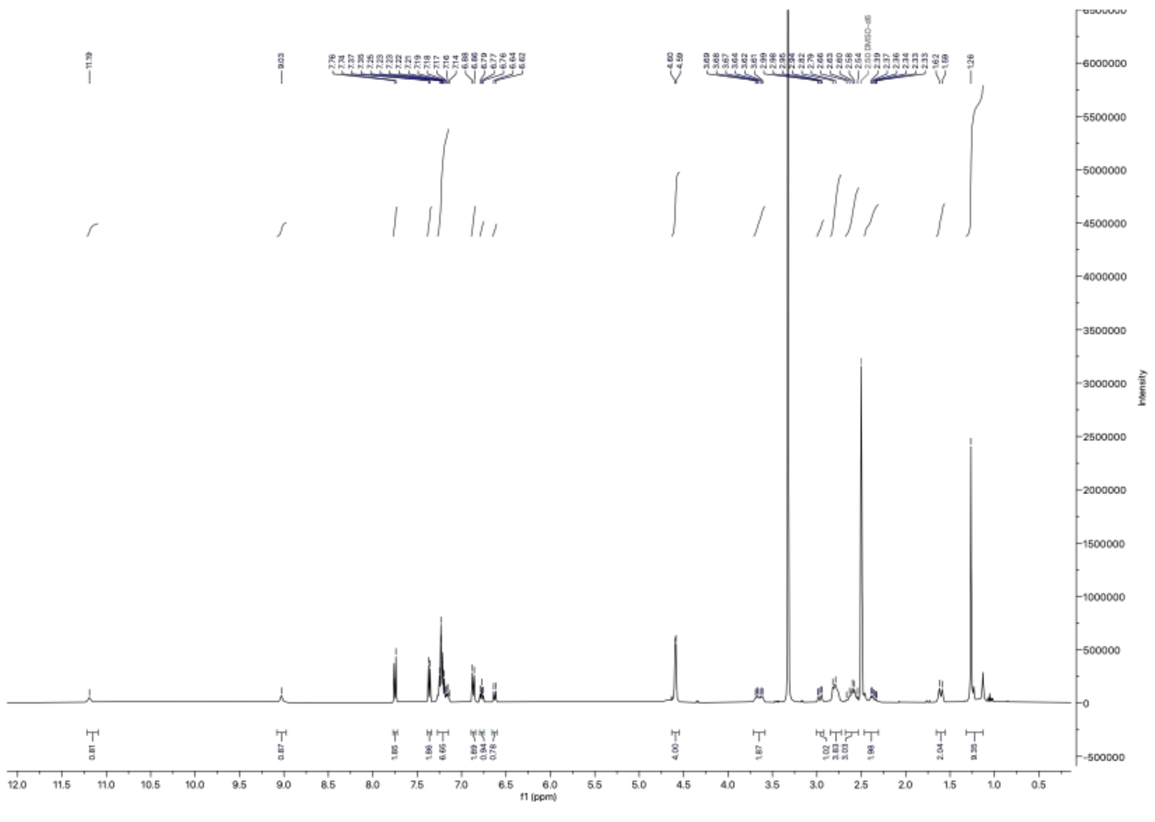

^{13}C NMR of 6 (F2F202)

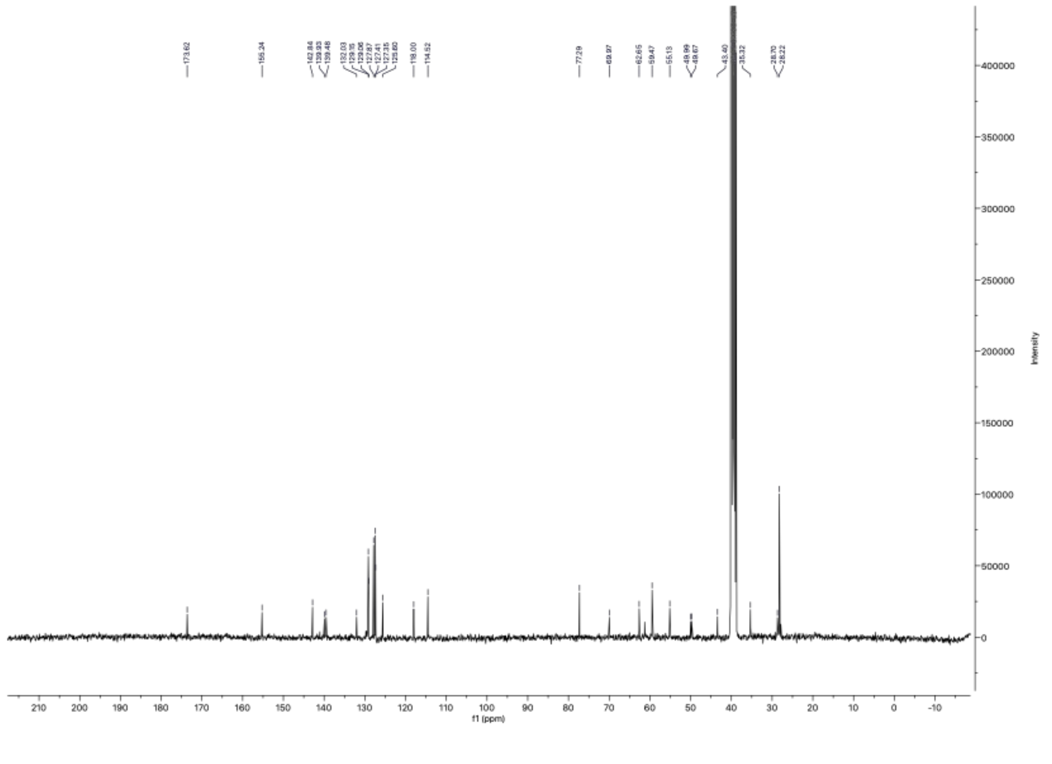

UPLC-MS spectra of compound 6 (F2F202)

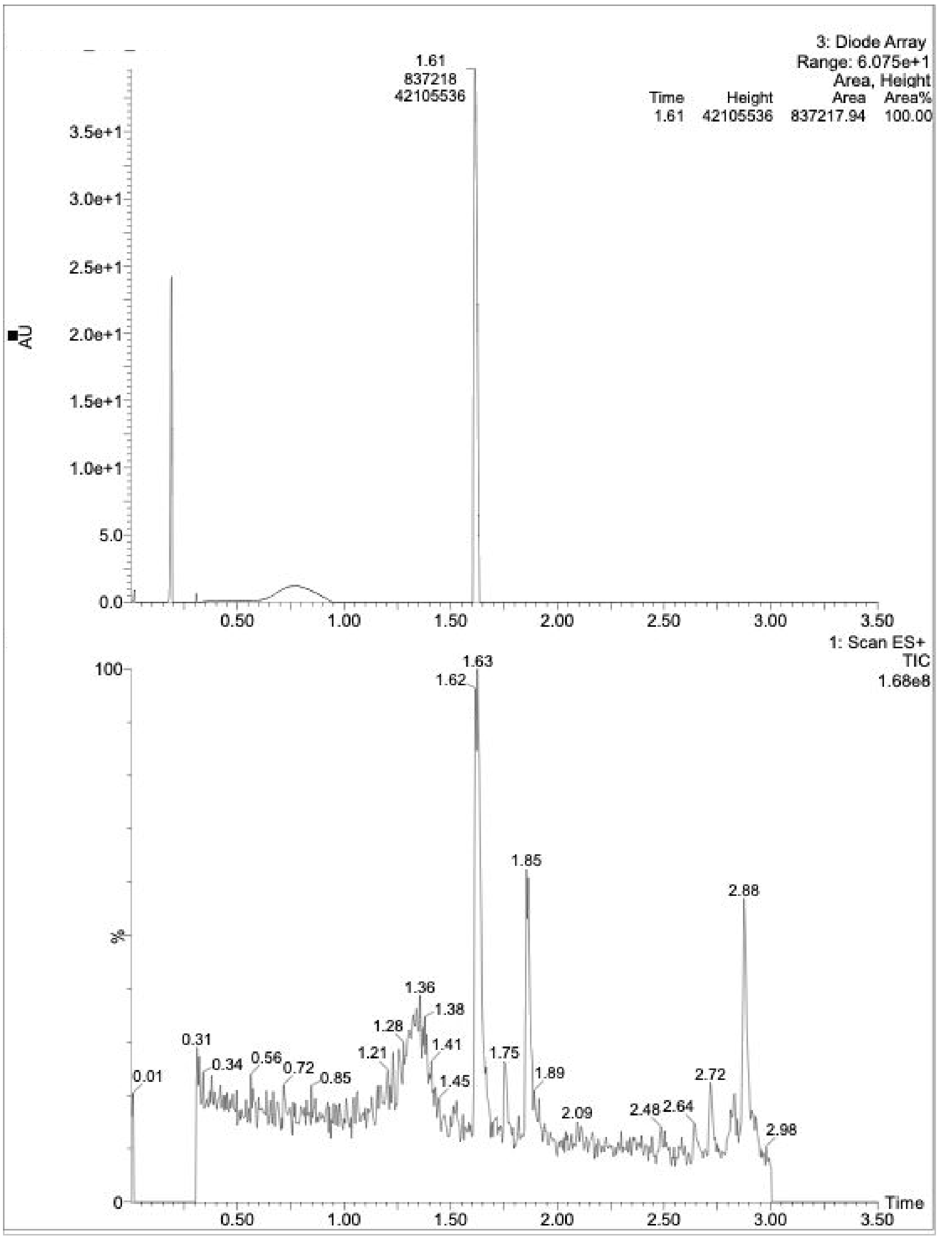

Experimental

Bioscreens *in vitro*. Biological effects in vitro of **6**, VRC (voriconazole) and the synergistic combination **6**/VRC (SYN) were investigated through the estimation of a "cell survival index", arising from the combination of cell viability evaluation with cell counting, as previously reported by us (Pharmaceutics. 2025;17(7):918). The cell survival index is calculated as the arithmetic mean between the percentage values derived from the MTT assay and the automated cell count. For this aim, human keratinocytes HaCaT (kindely provided by Valeria Cicatiello, Italian National Research Council (CNR), Institute of Genetics and Biophysics, Naples, Italy) were grown in DMEM supplemented with 10% FBS, L-glutamine (2 mM), penicillin (100 units/ml) and streptomycin (100 µg/mL) and were cultured in a humidified 5% carbon dioxide atmosphere at 37°C, according to supplier's recommendations. Cells were inoculated in 96-microwell culture plates at a density of 10^4 cells/well and allowed growing for 24 h. The medium was then replaced with fresh medium, and cells were treated for additional 24 and 48 h with VRC 0.125 µg/mL; 6 100 µM and SYN: 6 100 µM + VRC 0.125 µg/mL. DMSO, as vehicle for in vitro treatments, was used at a final biocompatible concentration of 0.25% in well. Cell viability was evaluated using the MTT assay procedure. Cell number was determined by TC20 automated cell counter (Bio-Rad, Milan, Italy), providing an accurate and reproducible total count of cells and a live/dead ratio in one step by a specific dye (trypan blue) exclusion assay. Data are expressed as percentages of untreated control cells and are reported as mean ± SEM (n = 4), by using a curve fitting program, GraphPad Prism 8.0.

Results. Bioscreen *in vitro* indicates a reduction in cell survival index after treatment with compound **6** at a concentration of 100 µM following 24 and 48 hours. No interference with cell survival was observed after application of VRC at a concentration of 0.125 µg/mL. The co-treatment (SYN) elicited cellular responses comparable to those of compound 6 alone.

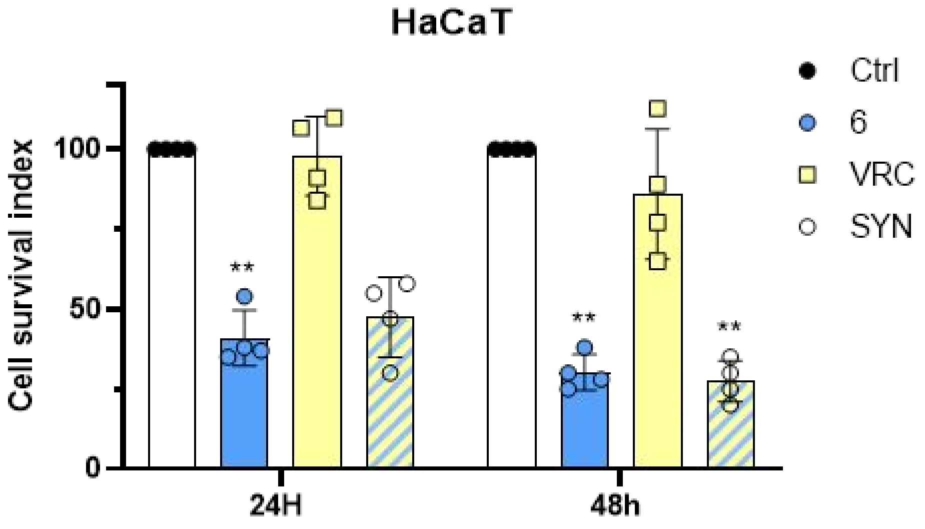

Figure S1. Cell survival index, evaluated by the MTT assay and live/dead cell ratio analysis, for human immortalized keratinocytes HaCaT following 24 and 48 h of incubation with **VRC** (voriconazole) 0.125 µg/mL; **6** 100 µM; **SYN** (Synergism): **6** 100 µM + **VRC** 0.125 µg/mL. Data in line graphs are expressed as percentages of untreated control cells and are reported as mean ± SEM (n = 4). ** $p < 0.01$ vs. control cells.
